# Postacute Sequelae of COVID (PASC or Long COVID): An Evidenced-Based Approach

**DOI:** 10.1093/ofid/ofae462

**Published:** 2024-08-27

**Authors:** Daniel O Griffin

**Affiliations:** Division of Infectious Diseases, Department of Medicine, Columbia University, College of Physicians and Surgeons, New York, New York, USA

**Keywords:** COVID-19, long COVID, PASC, postacute sequelae of COVID, SARS-CoV-2

## Abstract

While the acute manifestations of infectious diseases are well known, in some individuals, symptoms can either persist or appear after the acute period. Postviral fatigue syndromes are recognized with other viral infections and are described after coronavirus disease 2019 (COVID-19). We have a growing number of individuals with symptoms that persist for weeks, months, and years. Here, we share the evidence regarding the abnormalities associated with postacute sequelae of COVID-19 (PASC) and therapeutics. We describe physiological and biochemical abnormalities seen in individuals reporting PASC. We describe the several evidence-based interventions to offer patients. It is expected that this growing understanding of the mechanisms driving PASC and the benefits seen with certain therapeutics may not only lead to better outcomes for those with PASC but may also have the potential for understanding and treating other postinfectious sequelae.

## Teaser

A growing number of individuals suffer from Long COVID. This article describes the objective physiological and biochemical abnormalities seen in these individuals and the different clinical phenotypes of Long COVID. This article shares the evidence-based interventions available for Long COVID.

The first cases of a novel respiratory disease were described in China and then seen throughout the world [1, 2]. It was soon appreciated that this disease was, for a number of infected individuals, not simply a 1- or 2-week illness but one that could lead to longer-term suffering. Coronavirus disease 2019 (COVID-19), as this disease came to be known, turned out to be a disorder with a multitude of clinical presentations and a series of stages that could lead to a prolonged period of illness for many individuals [3]. While the occurrence of postinfectious sequelae with COVID-19 was initially met with surprise and dismissiveness by some, this phenomenon of ongoing or new-onset issues following infection is well described for many infectious diseases [4]. There is a considerable amount of literature describing a number of sequelae, including neurological disorders, psychiatric disorders, and debilitating fatigue after influenza, Ebola, diphtheria, polio, and other infections [5–10].

A growing literature describes the clinical features, the incidence in different populations, and the clinical, physiological, and biochemical alterations seen in individuals who do not fully recover after acute COVID-19 [11–14]. Many have focused on post-COVID-19 conditions (PCCs) in adults, but post-COVID-19 conditions are also seen in children and adolescents [15–17]. It has become clear that long COVID is seen in both patients who required hospitalization for acute COVID-19 and those who did not require hospitalization [18]. While many publications have listed different hypotheses regarding the possible mechanisms driving postacute sequelae of COVID-19 (PASC) and shared what people are trying, this article will share the evidence, or lack thereof, regarding the abnormalities associated with PASC and therapeutics.

## METHODS

In early 2020, the directors, staff, and contributors at Parasites Without Borders and Microbe TV, 2 New York–based nonprofits, started following the clinical presentations and literature surrounding COVID-19. These observations and discussions of the literature were initially shared on the podcast This Week in Virology (TWIV) and posted to the Parasites Without Borders website with links to the articles discussed. The articles for consideration on the podcast and website, and included in this review, were selected by the directors of Parasites Without Borders (Daniel Griffin, Dickson Despommier, Charles Knirsch, Vincent Racaniello, and Peter Hotez) and the directors of MicrobeTV (Vincent Racaniello, Daniel Griffin, Kathy Spindler, and Amy Rosenfeld). Listeners also suggested articles, which were included if the directors found them appropriate. Due to the very high number of articles being published, not all papers could be selected for discussion. When an article was selected, a literature review using key terms was conducted to identify similar articles for reference during the discussions and review of the articles. This article will follow the history of the growing appreciation of the phases of COVID-19 and the tail of manifestations that came to be recognized under the umbrella term PASC as presented each week. Articles identified and discussed as contributing to this growing understanding in the episodes released from February 2020 to June 1, 2024, are included. This article will focus on diagnosis of PASC, evidence regarding the mechanisms driving long COVID, challenges around estimating the incidence of long COVID, biochemical abnormalities, physiological abnormalities, long COVID phenotypes, prevention of long COVID, and therapeutics for long COVID. Estimates regarding the number of patients impacted suggest that this will be a disease for which primary care providers will often need to take the lead. Therefore, this article was written with the goal of preparing providers to make the diagnosis, provide treatment, and refer as needed [19].

## RESULTS

### The Stages of COVID-19: Recognition of Long COVID

Early articles and observations focused on the acute manifestations of COVID-19 and its different phases [3, 20–23]. By spring 2020, it became evident that not everyone infected with severe acute respiratory syndrome coronavirus 2 (SARS-CoV-2) was recovering during the weeks and months after infection, and an appreciation for long COVID began to emerge [3, 23]. The term “long COVID” was coined by the patients who were experiencing this prolonged illness [24, 25]. A number of individuals who had recovered from COVID-19 formed groups such as Survivor Corps. They thought their postinfection immunity would allow them to take care of the next wave of infected people. Instead, many of these survivors found that they were continuing to suffer and needed help themselves.

**Figure 1.**
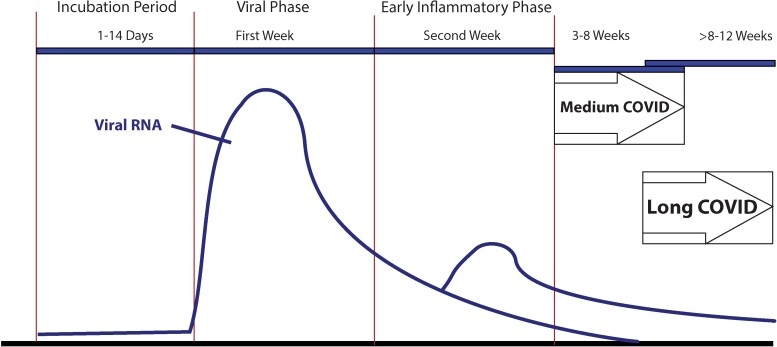
The stages of COVID-19: the incubation period, early inflammatory phase, delayed recovery period (medium COVID phase), and long COVID phase. Abbreviation: COVID-19, coronavirus disease 2019.

An inflammatory phase triggered after the period of viral replication was identified in the first few months of the COVID-19 pandemic, with an initial period described as a cytokine storm (Figure 1) [2]. While the initial viral replication would taper off quickly in about 7 days, this would be followed soon after by seroconversion [26, 27]. An association between elevated interleukin 6 (IL-6) and C-reactive protein (CRP) after this initial period of viral replication was soon correlated with risk of respiratory failure and mechanical ventilation [28]. This led to some of the first positive reports on the use of tocilizumab, a monoclonal that targets the IL-6 pathways, for the treatment of patients during this early inflammatory phase [29–34]. Soon after this, the results of the Randomized Evaluation of COVid-19 thERapY (RECOVERY) trial were released as a press release and later published, demonstrating the mortality benefit of low-dose dexamethasone after the first week and during the early inflammatory phase [35]. It came to be appreciated that both venous and thromboembolic complications were common problems in many hospitalized patients with COVID-19 [20, 22]. At this point, many have had multiple infections with SARS-CoV-2. Repeated infections with SARS-CoV-2 can increase the risk of postacute sequelae associated with COVID-19 [36, 37].

While many clinicians had been seeing patients in the early months of 2020 who were still reporting issues weeks and months after their acute infection, much of the discussion around the patient-coined terms long COVID and long-haulers was occurring on social media [24]. An article by Ed Yong, published on August 19, 2020, in *The Atlantic,* was, for many, the first time they had heard of long-haul COVID and the support groups that had arisen to help. The term “long-term COVID” may have first appeared on Twitter in May of 2020. By July 2020, descriptions of a chronic aftermath were being described in the medical literature in journals such as *JAMA* and *Science* [38, 39]. The term “medium COVID” was introduced to describe the experience of individuals who had ongoing issues after 2 weeks but had not reached the 8 or 12 weeks that many were using to make the diagnosis of long COVID. By the fall of 2020, reports of long COVID were noted to be rising, and it was clear that little was known about what appeared to be a new disease [40, 41]. January 4, 2021, was when long COVID became a regular topic in the Clinical Update section of our “This Week in Virology” weekly podcast and YouTube series.

It was becoming clear that the patients with ongoing symptoms often had involvement of many organ systems and seemed to be clustering into different long COVID groups or phenotypes [40, 41]. These different symptom clusters or phenotypes were associated with different persistent symptoms and different evolutions over time [42].

One encouraging message is that the majority of people have slowly decreasing symptoms, while only some having highly persistent symptoms [43]. The natural history of long COVID is such that most impacted individuals will improve, with only a minority still having a persistent condition at 2 years [43–46].

### Diagnosis of Postacute Sequelae of COVID

Patients themselves started using the terminology “long COVID” and “long-haul COVID.” Others introduced the terms “PASC” and “PCCs” to try to encompass the broader group of patients who were impacted. The World Health Organization (WHO) defines long COVID as the continuation or development of new symptoms 3 months after SARS-CoV-2 infection, with symptoms lasting for at least 2 months with no other explanation [47–51]. The US Centers for Disease Control and Prevention (CDC) defines long COVID as a chronic condition that occurs after SARS-CoV-2 infection and is present for at least 3 months [47–50, 52]. Long COVID is not a diagnosis of exclusion but is instead, like many diagnoses, one where there is a consistent history. In some cases, there are objective physiological abnormalities; in other cases, there are objective biochemical abnormalities; and in all cases, one needs to be careful not to attribute all issues to this one cause without a careful evaluation for other diagnoses. Long COVID requires a clinical diagnosis of acute COVID-19. However, a molecular test result is not required for the diagnosis.

### Possible Mechanisms Driving Long COVID

It is likely that different individuals have different underlying disturbances responsible for their ongoing illness. A number of ideas have emerged to explain the mechanism responsible for long COVID (Table 1). Many studies support ongoing immune activation or immune dysregulation driving many cases of long COVID [53]. A more sophisticated interpretation is the recognition of exhausted SARS-CoV-2-specific CD8+ T cells, a miscoordination between T-cell and B-cell responses, and improper crosstalk between the cellular and humoral adaptive response leading to immune dysregulation [54]. Part of the immune dysfunction appears to be persistent complement dysregulation and altered coagulation. This is marked by increased soluble C5bC6 complexes and decreased levels of C7-containing terminal complement complex (TCC) formations. These complexes and formations incorporate into cell membranes and are associated with elevated platelet activation markers and monocyte–platelet aggregates [55].

**Table 1.** Possible Mechanisms Driving Long COVID

Mechanism	Evidence	References
Ongoing immune activation/dysfunction	Exhausted SARS-CoV-2-specific CD8+ T cells Miscoordination between T-cell and B-cell responses Persistent complement dysregulation Altered coagulation marked by increased soluble C5bC6 complexes and decreased levels of C7-containing terminal complement complex formations Elevated platelet activation markers Monocyte–platelet aggregates	[53–55]
Remnant SARS-CoV-2 RNA	Detection of viral RNA using in situ hybridization Detection of viral RNA PCR amplification	[56–60]
Remnant SARS-CoV-2 protein	Detection of SARS-CoV-2 spike protein in biopsies	[57–59]
Dysbiosis	Depletion of certain microbes, such as *Bifidobacterium* Overgrowth of certain microbes, such as *Bacteroides, Clostridium,* and *Firmicutes*	[61, 62]
Endothelial dysfunction and dysfunction of the clotting system	Elevated platelet activation markers Monocyte–platelet aggregates	[55]
Neuroinflammation/blood–brain barrier dysfunction	Gliosis Microglia activation Polyclonal B-cell activation Exhausted T cells Decreased gray matter volume Impairment of the blood–brain barrier	[63–69]
Neural dysfunction (particularly vagal nerve dysfunction)	Thickening and hyperechogenic vagal nerves Reduced intestinal peristalsis Tachycardia Orthostatic hypotension Dizziness Dysphonia Dysphagia	[70]
Mitochondrial dysfunction	Downregulation of core mitochondrial genes Myopathy and focal necrosis triggered by postexertional malaise	[71–73]
Residual damage from acute infection	Pulmonary fibrosis Cardiac damage Hypoxemic damage to brain and other organs	[74–76]

Abbreviations: COVID, coronavirus disease 2019; PCR, polymerase chain reaction; SARS-CoV-2, severe acute respiratory syndrome coronavirus 2.

Several studies have indicated that the antigens of SARS-CoV-2, such as the proteins and RNA, can persist and drive ongoing immune abnormalities [56, 57]. There is a correlation between long COVID brain fog, muscle pain, and longer time to clear SARS-CoV-2 RNA from the upper respiratory tract during acute infection [58]. SARS-CoV-2 viral RNA has been detected in the liver, kidney, stomach, intestine, brain, blood vessels, lung, breast, skin, and thyroid for months after infection and may correlate with the development of long COVID symptoms [59]. While SARS-CoV-2 RNA has been detected in respiratory and nonrespiratory tissues for months after acute infection, there is limited evidence to support direct cytopathology outside the respiratory tract [60]. Despite the presence of SARS-CoV-2 RNA for months after infection, there has not been compelling evidence that replication-competent virus is present or that antiviral therapy in long COVID can impact symptoms.

A significant amount of evidence has established that there is a disturbance in the gut microbiome, leading to the suggestion that this is a significant factor driving the development of long COVID [61]. Metagenomic sequencing of the gut microbiome and microbial pathways has revealed depletion of certain microbes normally present in the healthy fecal microbiota of individuals with long COVID [61, 62].

Persistent complement dysregulation and altered coagulation have been identified. These are marked by increased soluble C5bC6 complexes and decreased levels of C7-containing terminal complement complex (TCC) formations. These complexes and formations incorporate into cell membranes and are associated with elevated platelet activation markers and monocyte–platelet aggregates [55]. It is proposed that this endothelial dysfunction and a disordered coagulation system may be driving disease in some individuals [55].

Acute blood biomarker profiles can predict cognitive deficits months after COVID-19 hospitalization, suggesting that inflammatory abnormalities may start during the acute period [63]. In attempts to understand the neurocognitive manifestations of postacute sequelae of COVID, some investigators have looked at mouse models and identified evidence of neuroinflammation and brain damage through multilineage neural cell regulation and axonal demyelination [64]. While ongoing inflammation may be a driver, gliosis consequent to inflammation in parts of the brain, such as the ventral striatum and dorsal putamen, may explain some of the depressive and cognitive symptoms [65]. There is also evidence of microglia activation, polyclonal B-cell activation, exhausted T cells, and impairment of the blood–brain barrier [66]. While research suggests that the loss of smell and taste seen with acute and postacute COVID results from impacts isolated to the sustentacular cells and alterations in the olfactory epithelium, some have identified decreased gray matter volume in individuals with compromised olfactory function [67, 68]. Studies using dynamic contrast-enhanced magnetic resonance imaging suggest a role for sustained systemic inflammation and localized blood–brain barrier (BBB) dysfunction as features of long COVID–associated brain fog [69].

Investigators have reported evidence of ongoing neural dysfunction, particularly involving the vagal nerve, in individuals with long COVID. This may explain symptoms such as cognitive dysfunction, gastroesophageal reflux, tachycardia, orthostatic hypotension, dizziness, dysphonia, dysphagia, and dyspnea [70]. Ultrasounds in some individuals show thickened and hyperechogenic vagal nerves along with reduced intestinal peristalsis [70].

Some attention has been given to mitochondrial dysfunction based partly on studies showing downregulation of core mitochondrial genes during acute COVID [71]. This downregulation of core mitochondrial genes has been suggested as a mechanism that may explain the triggering of postexertional malaise and the resulting myopathy and focal necrosis [72]. Mitochondrial dysfunction is also invoked to explain why, after certain types of exertion, some long COVID patients will develop postural orthostatic tachycardia, muscle pain, and cognitive issues [73].

One seemingly obvious explanation for long COVID-19 is the damage occurring during acute COVID-19 [74]. This is a compelling explanation for lung damage in patients who required mechanical ventilation [75]. Hypoxemia during acute illness also seems a likely contributor to ongoing cognitive issues [76]. Months in an intensive care unit would be expected to have impacts that would extend for some time after hospital discharge.

### Incidence of Postacute Sequelae of COVID-19 During the Pandemic

A significant challenge in understanding post-COVID-19 conditions and estimating the incidence is that there are several definitions advanced by different organizations such as the UK National Institute for Health and Care Excellence (NICE), the World Health Organization (WHO), the National Institutes of Health (NIH), and the US Centers for Disease Control and Prevention (CDC) [47–50]. A significant point of variation between the definitions has been the amount of time after acute COVID that symptoms must persist before patients are classified as having long COVID or a post-COVID condition [47–50, 77].

While much attention has focused on adults, PASC has been identified in children, adolescents, and university-aged individuals [78–80]. Long COVID is seen in health care workers, where there can be barriers to diagnosis and treatment [81]. Long COVID is not a condition limited to high-resource settings, with persistent postacute issues being seen in resource-limited regions [82, 83]. In several large commercial insurance databases, there is an increased incidence of certain conditions such as cardiac disorders, pulmonary disorders, and increased mortality over the 12 months after a diagnosis of acute COVID-19 [84].

Certain characteristics such as being hospitalized, female sex, higher body mass index, smoking, preexisting comorbidities, not receiving early antiviral therapy, and not being vaccinated are associated with an increased risk of long COVID. However, sequelae of COVID-19 are seen in all populations at varying incidence levels [85–88]. Certain genetic factors that influence the risk of developing long-term symptoms after acute COVID have been identified [89].

Long COVID has been seen since the early days of the pandemic and is still diagnosed after acute COVID. The incidence of long COVID varied during the pandemic, particularly in the prevaccine period [90]. The actual incidence is difficult to define as it is dependent on the definition used and the population being studied.

### Biochemical and Immunological Abnormalities

Detailed immune profiling identified some of the first objective biochemical and immunological abnormalities in post-COVID [14]. There is growing evidence that there is an immunopathogenic etiology to long COVID [91]. Not all of the testing is available to clinicians outside of a research setting. These differences are more consistent with a chronic inflammatory process than an auto-antibody-dominated disease process [14]. Persistently high levels of interferon-gamma that are CD8+ T-cell-mediated have been noted in patients with long COVID. The decrease of high levels of interferon-gamma to baseline levels correlates with symptom improvement (Table 2) [92].

**Table 2.** Biochemical and Immunological Abnormalities

Available for Routine Testing by Clinicians
Markedly elevated EBV serologies
Markedly elevated CMV serologies
Markedly elevated VZV serologies
Low a.m. serum cortisol without compensatory ACTH elevation
Anemia
Abnormal levels of serum iron
Lymphopenia
Thrombocytopenia
Diminished serum serotonin
Abnormal coagulation studies (PT/PTT, D-dimer)
Low albumin levels
Elevated serum aminotransferases (AST/ALT)
Abnormal lactate dehydrogenase levels
Elevated C-reactive protein
Elevated erythrocyte sedimentation rate
Elevated bilirubin levels
Elevated lipase levels
Available in research settings
Elevated levels of neurofilament light chain
Depletion of commensal bacteria
Abnormal activation of CMV-specific CD8+ cells
Viral antigen persistence

Abbreviations: ACTH, adrenocorticotropic hormone; AST, aspartate aminotransferase; ALT, alanine transferase; CMV, cytomegalovirus; EBV, Epstein-Barr virus; PT, prothombin; PTT, partial thromboplastin time; VZV, varicella-zoster virus.

There is evidence to suggest that disturbance in the gut microbiome, gut microbiome dysbiosis, is a significant factor driving long COVID in some of the different phenotypic manifestations [61]. Metagenomic sequencing of the gut microbiome and microbial pathways has revealed that depletion of *Bifidobacterium adolescentis* and *Roseburia hominis* and enrichment of *Clostridium bolteae* and *Flavonifractor plautii* are seen in the fecal microbiota of individuals with long COVID. Three specific gut enterotypes associate with individual symptoms [61].

Investigators have identified laboratory abnormalities that can be measured in the clinic, such as significantly higher antibody levels against non-SARS-CoV-2 viral pathogens, particularly Epstein-Barr virus (EBV), cytomegalovirus (CMV), and varicella-zoster virus [14]. Investigators have noted low cortisol levels that are not associated with a compensatory increase in adrenocorticotropic hormone (ACTH) levels [14]. Repressed levels of cortisol during acute COVID have been detected [80]. At the time of initial diagnosis, EBV viremia is an early anticipating risk factor and potentially a causative phenomenon that may drive the development of long COVID [80, 91]. Viremia of reactivated latent viruses, along with abnormalities of activation of CMV-specific CD8+ T cells, has been detected [80]. While there may be EBV viremia during acute COVID and there is evidence of very elevated EBV serologies in patients with long COVID, ongoing EBV activation does not appear to be present [93].

While some investigations have detected viral antigen persistence, such as SARS-CoV-2 spike protein and SARS-CoV-RNA, in tissues and fecal samples, it is unclear if this is driving disease or merely a correlate. There has been no significant success demonstrating persistent replication-competent virus in patients with post-COVID conditions despite many investigations [94]. Some investigators have identified combinations of inflammatory mediators that can be used to identify long COVID with a high degree of accuracy [95]. Associations between individual symptoms of postacute sequelae and certain T-cell subsets have been identified [96]. The ongoing inflammatory process may explain anemia, low serum iron, altered iron homeostasis gene expression, pronounced iron-deficient reticulocytosis, lymphopenia, low numbers of dendritic cells, iron maldistribution, and emerging stress erythropoiesis [97].

In a subset of patients, circulating serotonin levels are reduced, potentially driven by viral RNA-induced type I interferons, diminished tryptophan uptake, and decreased serotonin storage secondary to thrombocytopenia [98]. It has been suggested that this serotonin deficiency impairs cognition via reduced vagal signaling [98].

There is a significant increase in abnormal coagulation and liver and biliary-related serum tests. Low albumin levels, elevated serum aminotransferases, elevated lactate dehydrogenase, elevated C-reactive protein, elevated bilirubin, and elevated lipase levels have been detected [99].

Elevated levels of neurofilament light chain in COVID-19 patients with ongoing neurocognitive symptoms post-COVID suggest that neuroaxonal damage may be present. These elevated levels may correlate with cognitive loss and long COVID exacerbations [100].

### Physiological Abnormalities

Several cardiovascular complications with objective physiological abnormalities are seen in the postacute period. Cardiac MR (CMR) abnormalities have been detected in individuals with long COVID for months following acute infection [101]. There is a high incidence of autonomic dysfunction and postural orthostatic tachycardia syndrome (POTS) in patients with PASC [102]. These have been documented in pediatric patients as well as adults [103]. Autonomic dysfunction can be documented with formal testing using the 10-minute active standing test, the National Aeronautics and Space Administration Lean Test (NLT), and sudomotor assessment testing [102]. An exaggerated orthostatic blood pressure response (EOBP) can be detected using a heads-up tilt test (HUTT) [104]. Autonomic dysregulation in patients with long COVID can be assessed by measuring heart rate variability with 24-hour Holter-ECG monitoring [105]. While not all patients suffering from long COVID are aware of orthostatic intolerance (OI), screening patients with a standardized NLT can identify patients with OI as well as those with unrecognized POTS and lead to treatment [106]. Measuring heart rate recovery at the first minute is another way to assess the impact of long COVID and may guide some therapeutics [107]. Formal cardiopulmonary exercise testing (CPET) can document reduced VO2 peak, abnormal cardiovascular efficiency, pathological VE/VCO, and abnormally reduced slope of VO2 work (Table 3) [108].

**Table 3.** Physiological Abnormalities

Abnormal Test	How to Perform or Order	References
Abnormal NASA Lean Test	After measuring baseline blood pressure and heart rate supine, the patient stands with heels about 6–8 inches from a wall and relaxes and leans against the wall. Record blood pressure, heart rate, and symptoms every minute for 10 minutes (abnormal if heart rate increases >30 beats per minute or rate increases to >120, a decrease in systolic blood pressure of >20, or a decrease in diastolic blood pressure of >10).	[102]
Abnormal Sudomotor Assessment	Diagnostic machines are used to perform the QSART or sweat test using electrodes that assess the integrity of small unmyelinated postganglionic sympathetic sudomotor C-fibers. This test may require referral to a practice that provides this testing.	[102]
Exaggerated orthostatic blood pressure response on heads-up tilt test	This test is performed at centers with a special table that tilts patients from 0°–30°–45°–70° and records heart rate, blood pressure, and symptoms. Tests are interpreted and results provided for the ordering clinician.	[104]
Heart rate variability on 24-hour Holter-ECG monitoring	Obtaining Holter monitoring may require referral to a cardiologist, but in some areas testing can be ordered by primary care providers with provided interpretations.	[105]
Abnormal heart rate recovery at 1 minute postexertion	Measuring heart rate at 1 minute postexertion measures the fast phase of recovery; normal is a drop of >18 beats per minute by the first minute postexertion.	[107]
Abnormal cardiopulmonary exercise testing results	Cardiopulmonary exercise testing may require a referral to a center or office that uses specialized equipment such as a treadmill or bicycle and ECG monitoring, oximetry monitoring, or a mask with flow monitoring.	[108]
Dysrhythmias detected on ECG monitoring	Short in office rhythm strips and Holter monitoring can detect dysrhythmia.	[109, 110]
Abnormal pulmonary function testing with inclusion of DLCO testing	While chest radiographs and basic pulmonary function testing can be normal, more abnormalities can be detected with inclusion of DLCO testing.	[111]

Abbreviations: DLCO, diffusing capacity of the lungs for carbon monoxide; ECG, electrocardiogram.

There is a significant excess burden of dysrhythmias, including atrial fibrillation, atrial flutter, sinus tachycardia, sinus bradycardia, and ventricular arrhythmias [109, 110]. As part of the sequelae of COVID-19, individuals may also develop pericarditis, acute coronary disease, heart failure, cardiovascular disorders such as strokes and transient ischemic attacks (TIAs), and thrombotic disorders including deep vein thrombosis, superficial vein thrombosis, and pulmonary emboli [109, 112].

The impact of long COVID on the lungs can be detected with chest computed tomography (CT) and pulmonary function testing with measurement of diffusion capacity. Such testing can detect diffusion impairment (diffusing capacity of the lungs for carbon monoxide [DLCO] ≤80%) and pulmonary restriction (TLC ≤80%), as well as CT evidence of pulmonary fibrosis in individuals with dyspnea as a dominant symptom [111]. An important consideration is that the pulmonary exam and simple radiographs, as well as more advanced testing, can be unremarkable in many patients with long COVID despite symptoms.

### Long COVID Phenotypes

Acute COVID-19 can significantly increase the risk of death in the 6 months after acute infection. Following COVID-19, there is a significantly higher incidence of new respiratory conditions, cardiovascular disease, neurological issues, stroke, encephalopathy, dementia, and diabetes [113–117]. The recognized overlapping and often shifting phenotypes of long COVID can be identified by analysis of self-reported symptoms. Some investigations have suggested that long COVID patients can be clustered into 3 different groups that might be driven by distinct underlying pathophysiologic mechanisms of disease [118]. Cluster 1 had predominantly pain symptoms with a higher proportion of joint pain, myalgia, and headache; cluster 2 had a preponderance of cardiovascular symptoms with prominent chest pain, shortness of breath, and palpitations; and cluster 3 had significantly fewer symptoms than the other clusters [118]. Other studies have suggested that there may be more phenotypic clusters or have focused on the impacts on different organs (Table 4) [13, 119, 120].

**Table 4.** Long COVID Clinical Phenotypes

Phenotype	Features
Myalgic encephalitis/chronic fatigue syndrome phenotype	A substantial reduction or impairment in the ability to engage in pre-illness levels of activity that lasts for >6 months Fatigue lasting >6 months that is profound, of new onset, not the result of ongoing or unusual excessive exertion, and not substantially improved with rest Postexertional malaise and or unrefreshing sleep Cognitive impairment Orthostatic intolerance
Cardiovascular phenotype	Chest pain, palpations, atrial fibrillation, atrial flutter, sinus tachycardia, sinus bradycardia, and/or ventricular arrhythmias Impaired myocardial flow reserve The new diagnosis of heart failure or myo/pericarditis
Respiratory phenotype	Difficulty breathing with exertion New diagnosis of asthma, bronchiectasis, COPD, pulmonary fibrosis, and lung cancer
Endocrine phenotype	Post-COVID adrenal insufficiency disorder with low cortisol levels without a compensatory ACTH response New diagnosis of type 2 diabetes or thyroid disease
Neurological phenotype	Difficulty concentrating and maintaining attention Memory issues Anosmia Dysgeusia Headaches Sleep disorders Neuropathies Cognitive dysfunction (often described as brain fog)
Gastrointestinal phenotype	Diarrhea, dyspepsia, bloating, distension, constipation, and irritable bowel–like presentations
Reproductive phenotype	Reduced sperm count Erectile dysfunction Irregular menstruation Increased severity of premenstrual symptoms
Allergic immune activation phenotype	Mast-cell activation–like syndrome presentations New diagnosis of rheumatological disorders New diagnosis of asthma or allergic rhinitis

Abbreviations: ACTH, adrenocorticotropic hormone; COPD, chronic obstructive pulmonary disease; COVID, coronavirus disease 2019.

Post-COVID conditions can be classified based on organ system involvement, looking at impacts on the heart, the lungs, the pancreas, the immune system, the gastrointestinal tract, the neurological system, the kidneys, the spleen, the liver, the blood vessels, or the reproductive system [13]. Immunophenotyping studies have detected different immunological endotypes based on analysis of immune cells, cytokines, antibodies, and viral signatures [80]. Certain proteomic unsupervised analyses can cluster certain patients into those with and those without inflammatory signatures [121].

Perhaps a more intuitive breakdown of phenotypes for patients and clinicians is into clinical clusters where individuals tend to group with not just 1 organ system involved but instead a cluster of symptoms. Along this line, there are several clusters described, including but not limited to a myalgic encephalitis/chronic fatigue syndrome phenotype, a cardiovascular phenotype, a respiratory phenotype, an endocrine phenotype, a neurological phenotype, a gastrointestinal phenotype, a reproductive system phenotype, and an allergic immune activation phenotype [19, 122–125]. It is important to recognize that the manifestations of long COVID are not static and can evolve over time, with substantial switching between phenotypes for individual patients, reinforcing the dynamic nature of post-COVID conditions [126].

### Myalgic Encephalitis/Chronic Fatigue Syndrome Phenotype

Among the recognized phenotypes is one where patients meet the criteria established for the diagnosis of myalgic encephalitis/chronic fatigue syndrome (ME/CFS). In line with the 2015 Institute of Medicine (IOM) diagnostic criteria, these individuals suffer a substantial reduction or impairment in the ability to engage in pre-illness levels of activity that lasts for >6 months and is accompanied by fatigue lasting >6 months that is often profound, of new onset, not the result of ongoing or unusual excessive exertion, and not substantially improved with rest, postexertional malaise, and or unrefreshing sleep. They also have cognitive impairment (impacting thinking, memory, executive function, information processing, attention, and psychomotor function) and orthostatic intolerance. These individuals may also suffer from the physical symptoms of muscle pain, polyarthralgia, sore throat, tender lymph nodes, or new-onset headaches [127]. Individuals in this cluster, as well as those who might be classified in the cardiovascular cluster, may report as well as have documented autonomic dysfunction such as POTS [13, 102]. The debilitating fatigue that many experience as part of the sequelae of COVID-19 is often the main symptom preventing those suffering from active participation in their activities of daily living [128, 129].

### Cardiovascular Phenotype

Individuals who fall into the cardiovascular phenotype may report chest pain, palpations, atrial fibrillation, atrial flutter, sinus tachycardia, sinus bradycardia, and/or ventricular arrhythmias [109, 130]. As part of the sequelae of COVID-19, individuals may also develop pericarditis, acute coronary disease, or new-onset heart failure [109, 131]. Patients classified in the cardiovascular cluster may report autonomic dysfunction, such as POTS. In addition to abnormalities seen with NASA lean testing and tilt-table testing, there is evidence of endothelial dysfunction with impaired myocardial flow reserve (MFR), calculated as the ratio of stress to rest myocardial blood flow (MBF) in mL/min/g of the left ventricle [132]. There is also an increased risk of new-onset persistent hypertension seen after acute COVID-19 [133]. While patients with autonomic dysfunction might be similar to those in the ME/CFS phenotype, there are a number of individuals with unrecognized autonomic dysfunction that can be identified through the use of the Composite Autonomic Symptom Score–31 (COMPASS31) or Work Ability Index questionnaires who experience a significant reduction in their ability to work [134].

### Respiratory Phenotype

The respiratory phenotype is typically characterized by reports of difficulty breathing with exertion but may also include new diagnosis of asthma, bronchiectasis, chronic obstructive pulmonary disease, pulmonary fibrosis, or lung cancer [125, 135]. While pulmonary exam and simple chest radiographs may not detect abnormalities in a number of patients with long COVID, chest CT and pulmonary function testing with measurement of diffusion capacity can detect diffusion impairment (DLCO ≤80%) and pulmonary restriction (TLC ≤80%) in a significant number of individuals with dyspnea as a dominant symptom [111].

### Endocrine Phenotype

An endocrine phenotype is also described. A post-COVID adrenal insufficiency disorder with low cortisol levels without a compensatory ACTH response has been observed [14]. There can be impacts on thyroid function and an increased incidence of type 2 diabetes [115, 136, 137].

### Neurological Phenotype

While many of the phenotypes are not stable, and there can be significant overlap between them, a neurological phenotype with cognitive impairment that can be documented on formal testing has been recognized [130, 138, 139]. The neurological phenotype is characterized by difficulty concentrating and maintaining attention, memory issues, anosmia, dysgeusia, headaches, sleep disorders, neuropathies, cognitive dysfunction (often described as brain fog), and headaches [80]. For some, chronic pain is the most significant symptom [125]. Short web-based cognitive tasks, such as Simple Reaction Time (SRT) and the Number Vigilance Test (NVT), as well as the Montreal Cognitive Assessment (MoCA), can be used to differentiate patients with the cognitive slowing seen in long COVID from those who recover [140, 141]. Documented memory problems are seen in individuals after COVID when subjected to Everyday Memory Questionnaire (EMQ) testing [142]. Cognitive impairment can be documented with the Addenbrooke's Cognitive Examination III (ACE III) screening test, an open-access alternative to the Mini-Mental Status Test (MMSE). Using formal cognitive testing, investigators have found that about half of patients with long COVID have episodic memory deficits, and about one-quarter also have impaired overall cognitive function, especially attention, working memory, processing speed, and verbal fluency [143]. Formal testing quantified this deficit as commensurate with a 3-point loss in intelligence quotient (IQ) in participants who had had mild COVID-19 with resolved symptoms. Larger deficits are seen in participants with unresolved persistent symptoms equivalent to a 6-point loss in IQ. Those who had been admitted to the intensive care unit had the equivalent of a 9-point loss in IQ. More significant deficits are seen in participants who had SARS-CoV-2 infection during periods in which the original virus or the B.1.1.7 variant was predominant. Greater cognitive impairment is also seen in individuals who had been hospitalized than in those who had not been hospitalized [144]. The cognitive profile of patients with the neurological phenotype of long COVID has been found to be similar to that of patients who have multiple sclerosis (MS), with similar impacts on attention and processing speed. There is more impairment in episodic memory in MS but more severe fatigue in long COVID [145].

### Gastrointestinal Phenotype

A gastrointestinal phenotype of long COVID has been identified with diarrhea, dyspepsia, bloating, distension, constipation, and irritable bowel-like presentations [99, 146]. Some people with this phenotype have documented intestinal methanogen overgrowth due to methane-producing archaea in the small intestine and carbohydrate malabsorption [146].

### Genitourinary or Reproductive Phenotype

Very troubling for many suffering from postacute sequelae of COVID are issues such as reduced sperm count, erectile dysfunction, irregular menstruation, and increased severity and number of premenstrual symptoms that suggest clustering into a genitourinary or reproductive phenotype [13]. It became evident during the first year of the pandemic, before the availability of COVID-19 vaccines, that COVID-19 infections were associated with a significantly increased risk of new-onset erectile dysfunction [147]. There is a demonstrated increase in the incidence of urinary retention, hematuria, and urinary tract infections after acute COVID that is seen regardless of COVID-19 severity in men and attributed to prostatic hyperplasia deterioration [148]. Semen quality can be significantly reduced after SARS-CoV-2 infection, with decreased sperm count and decreased motility [149]. Many of the impacts on reproductive health, such as menstrual irregularity, symptom exacerbation around menstruation, dyspareunia, infertility, pelvic congestion syndrome, adverse pregnancy outcomes, and endometriosis, have impacted women [150].

### Allergic Immune Activation Phenotype

Some report symptoms suggesting appropriate classification in the allergic immune activation phenotype category. Some individuals report symptoms that are suggestive of a mast cell activation syndrome [13]. There is an elevated risk of a number of rheumatological disorders developing after acute COVID, including but not limited to rheumatoid arthritis, ankylosing spondylitis, systemic lupus erythematosus, dermatopolymyositis, systemic sclerosis, Sjögren's syndrome, mixed connective tissue disease, Behçet's disease, polymyalgia rheumatica, vasculitis, psoriasis, inflammatory bowel disease, celiac disease, Graves’ disease, antiphospholipid antibody syndrome, pemphigoid, immune-mediated thrombocytopenia, multiple sclerosis, and type 1 diabetes [151–154]. Some individuals with an immune phenotype of post-COVID develop new-onset asthma or allergic rhinitis [155].

### Prevention of Long COVID

Not getting COVID is the best way to avoid getting long COVID. Behavior modifications, masking, and improved ventilation can reduce the risk of exposure and risk of getting COVID-19 [156]. Vaccines decrease the risk of developing long COVID in adults as well as adolescents and children [157–165]. One investigation that looked at individuals in Sweden reported that vaccine effectiveness against PCCs for 1 dose, 2 doses, and 3 or more doses was 21%, 59%, and 73%, respectively [166]. The risk of certain diseases, such as diabetes, after COVID-19 infection is reduced with vaccination pre-infection [167, 168]. Vaccination decreases the elevated risk of cardiovascular disease after COVID-19 [169]. Booster vaccinations may further reduce the risk of long COVID (Table 5) [170].

**Table 5.** Prevention of Long COVID

Methods	Evidence	References
Avoiding infection	Behavior modifications, masking, and improved ventilation prevent infection	[155]
Vaccination	Suggested vaccine effectiveness against PCCs for 1 dose, 2 doses, and 3 or more doses was 21%, 59%, and 73%, respectively The risk of certain diseases, such as diabetes, after COVID-19 infection is reduced with vaccination pre-infection Vaccination decreases the elevated risk of cardiovascular disease after COVID-19 Booster vaccinations may further reduce the risk of long COVID	[156–169]
Nirmatrelvir-ritonavir	Early treatment with nirmatrelvir-ritonavir is associated with a reduction in postacute incidence of congestive heart failure, atrial fibrillation, coronary artery disease, chronic pulmonary disease, acute respiratory distress syndrome, interstitial lung disease, and end-stage renal disease	[171–174]
Convalescent plasma	Early administration of COVID-19 convalescent plasma has demonstrated a reduction in cytokine levels and lower odds of development of post-COVID conditions	[175]
Corticosteroids	Corticosteroids may exert a protective effect against the development of post-COVID-19 syndromes if administered appropriately to hospitalized patients with acute COVID	[176]

Abbreviations: COVID-19, coronavirus disease 2019; PCCs, post-COVID-19 conditions.

There has been some excitement about the possible impact of early use of metformin on the risk of developing long COVID, but this required a challenging regimen in the published trial and has not been repeated [177]. There is also uncertainty as to whether early treatment with effective antivirals such as nirmatrelvir or molnupiravir may reduce the risk of all post-COVID conditions in people who are vaccinated, unvaccinated, boosted, and who have primary and SARS-CoV-2 reinfection [86, 171, 172, 178, 179]. Multiple studies have demonstrated that treatment of acute COVID-19 with nirmatrelvir-ritonavir can reduce the incidence of major adverse cardiac events (MACEs; cardiovascular death, myocardial infarction, stroke, new-onset heart failure, heart failure hospitalization, or ventricular arrhythmia) [173, 174]. In addition to a reduction in MACEs during the acute period, early treatment with nirmatrelvir-ritonavir reduces the postacute incidence of congestive heart failure, atrial fibrillation, coronary artery disease, chronic pulmonary disease, interstitial lung disease, and end-stage renal disease [172, 180].

Despite compelling data that monoclonal antibody therapy can have dramatic impacts on acute COVID-19 outcomes, our investigations looking at the ability of early monoclonal antibody therapy to prevent long COVID have not been encouraging [175]. While studies of the administration of monoclonal antibody therapy have not demonstrated a reduction in the development of long-term COVID, early administration of COVID-19 convalescent plasma (CCP) has demonstrated a reduction in cytokine levels and lower odds of development of post-COVID conditions [176]. Corticosteroids may exert a protective effect against the development of post-COVID-19 syndromes if administered appropriately to hospitalized patients with acute COVID [181].

### Therapeutics for Long COVID

One can open a discussion of evidence-based therapeutics for long COVID-19 with optimism, as there are hundreds of ongoing clinical trials for long COVID-19, the majority of which test potential therapies [182].

Before its consideration as a therapeutic, there were questions about the safety of vaccinating patients with long COVID symptoms. This was followed by evidence that COVID-19 vaccines have therapeutic benefits for patients with long COVID if given after the development of post-COVID sequelae [159, 161, 183–185]. Vaccination has also been shown to reduce the severity and impact of long COVID on patients’ social, professional, and family lives [186]. The benefits of COVID-19 vaccines on long COVID appear with a single vaccine dose but increase with a second dose and increase even more with a third dose of vaccine [183]. COVID-19 vaccination in the context of PCCs is associated with reduced symptoms, increased well-being, and downregulation of systemic markers of inflammation [187].

Sleep disturbances are reported at high prevalence rates after acute COVID, with some observations suggesting a higher prevalence associated with Black race and hospitalization for COVID-19 [188]. Evidence has suggested that melatonin may offer some benefit for individuals with PASC. Initially, melatonin was being used to address sleep disturbance issues reported by patients, but observations and then publications supported a potentially broader role for melatonin in long COVID patients through possible impacts on inflammation, clinical signs and symptoms, and recovery time [189].

Researchers have documented temporal changes in the fecal microbiota of patients infected with COVID-19 primarily characterized by increased *Bacteroides* and decreased *Prevotella*, with a negative correlation between disease severity and butyrate-producing bacteria such as *Lachnospiraceae*, *Roseburia,* and *Faecalibacterium* [190]. Studies demonstrating depletion of *Bifidobacterium adolescentis* and *Roseburia hominis,* enrichment of *Clostridium bolteae* and *Flavonifractor plautii,* and specific gut enterotypes associated with individual symptoms have suggested that attempts to modify the gut microbiome may impact long COVID [61]. Following evidence that acute COVID-19 can disrupt the gut microbiome, investigators studied the impact of prebiotics and probiotics on PASC. Promising results using probiotics containing bifidobacteria strains reduced nasopharyngeal viral load, reduced pro-inflammatory immune markers, and restored a healthy gut microbiome [191]. Results of a randomized, double-blind, placebo-controlled trial using a proprietary formulation (SUM01)—containing 3 bacterial strains, *Bifidobacterium adolescentis, Bifidobacterium bifidum,* and *Bifidobacterium longum,* and 3 prebiotic compounds, galacto-oligosaccharides, xylo-oligosaccharides, and resistant dextrin—that has been shown to promote the growth of these bacterial strains at a dose of 10 billion colony-forming units in sachets twice daily showed alleviation of fatigue, reduction in memory loss, improved concentration, reduction in gastrointestinal symptoms, and less general unwellness compared with the placebo group [192].

Some patients with long COVID tolerate and benefit from exercise. In contrast, a number of patients with long COVID develop postexertional malaise (PEM) and can be harmed without careful attention and broad recommendations for graded exercise training (GET). Much literature and controversy surround GET and pacing. There are concerns around any benefits of GET as well as potential harms for those with PASC. There is some evidence that, in a properly selected subset of individuals with long COVID without PEM, there can be some benefits to GET [193]. There is concern that investigations supporting pacing or GET have been poorly designed and just selected patients able to participate rather than proving a general benefit [194]. There is some evidence that inducing postexertional malaise can be harmful, triggering metabolic disturbances, myopathy, infiltration of amyloid-containing deposits, and focal necrosis [72]. Following high-intensity training (HIIT), moderate-intensity training (MICT), or strength training (ST), some long COVID patients will develop postural orthostatic tachycardia, increased muscle pain, and difficulty concentrating [73]. Cautious exercise adoption may prevent skeletal and muscle deconditioning but should be individualized for each patient and recommended only after screening for PEM to avoid potential harm. Measuring and then following heart rate recovery at the first minute after exercise termination can be useful in identifying individuals who might benefit from an inspiratory muscle training program and may allow for monitoring of response [107, 195]. Other monitoring approaches allow the use of smartphone-based apps [196].

Active cycle breathing techniques, such as box breathing, have been recommended to patients suffering from long COVID as studies have identified lower parasympathetic tone and evidence of autonomic dysfunction in these patients [197]. Slow breathing techniques have been shown to reduce heart rate and trigger vagal nerve activity [198]. In the context of long COVID, slow-paced breathing may modulate autonomic tone, resulting in decreased systolic blood pressure oscillation, heart rate variability, and improvement in symptoms [199].

Cognitive behavioral therapy (CBT) for severe fatigue following COVID-19 is being studied but remains controversial, with concerns about study design and a history of recommending this therapy for ME/CFS despite a lack of compelling evidence [200, 201]. There is a genuine concern that broad recommendations for CBT send a dismissive message. The ME/CFS literature does not support any significant or sustained benefit of CBT, and recommending CBT is contradictory to NICE ME/CFS guidelines [202].

Case reports have suggested that some therapies, such as immunoglobulin therapy, may provide benefits for some suffering from long COVID [203]. For individuals with impacts on working memory and executive function of the prefrontal cortex, case studies have suggested a potential benefit to treatment with the alpha-2A-adrenoreceptor agonist guanfacine (1 mg orally at bedtime for the first month, increased to 2 mg after 1 month if well tolerated) and the antioxidant N-acetylcysteine 600 mg daily [204]. There is evidence that antihistamines or low-histamine diets may provide some benefit for individuals suffering from long COVID [205]. This intervention may be more applicable to patients with an immune or allergic phenotype. Some case reports and investigations suggest benefit with combinations of H1 and H2 antihistamines [206–208].

Reduced circulating serotonin levels have suggested the use of higher-dose serotonin reuptake inhibitors or the use of alternative agents such as serotonin-norepinephrine reuptake inhibitors or norepinephrine-dopamine reuptake inhibitors [209]. The finding of low-serum serotonin has led to a trial using increased tryptophan ingestion through diet or supplements [98].

Olfactory dysfunction is a common manifestation of acute COVID-19, and continued olfactory dysfunction can significantly impact quality of life. There are limited interventions beyond time that can impact this sequela. Some findings suggest that topical nasal steroids may be beneficial, while systemic steroids do not seem to provide benefit [210].

Hyperbaric oxygen therapy has been tried for long COVID, with some suggestions that this might be associated with short- and longer-term impacts on quality of life, quality of sleep, and pain symptoms [211, 212]. These data are still limited for hyperbaric oxygen therapy, and it remains unclear whether this will be a therapy that benefits individuals with PASC.

Based on the idea that remnant viral RNA might be driving ongoing issues with long COVID, investigators studied treatment with catalytically active human RNase fused to human immunoglobulin G [213]. While the impact on fatigue was not statistically significant, it does represent an interesting approach based on ideas about the underlying mechanism driving long COVID.

A number of therapies are being actively investigated for long COVID treatment. This list and our knowledge of the best candidates for different interventions will only grow as we complete the investigations (Table 6) [214].

**Table 6.** Individualized Potential Therapeutics for Long COVID

Symptoms	Intervention	References
Considered for all patients	Vaccination after infection (evidence that this can reduce symptoms, increase well-being, and downregulate systemic markers of inflammation)	[158, 160, 182–186]
Patients with sleep disturbances, ongoing inflammation	Melatonin	[187, 188]
Patients with fatigue, memory loss, impaired concentration, gastrointestinal symptoms	Bifidobacteria containing probiotics	[189–191]
Patients without postexertional malaise	Cautious exercise adoption may prevent skeletal and muscle deconditioning but must be individualized for each patient	[72, 73, 107, 192–195]
Patients with decreased vagal tone as evidenced by increased blood pressure, heart rate variability, or anxiousness	Active cycle of breathing techniques (box breathing)	[98, 196–198]
Patients with impacts on working memory and executive function	N-acetylcysteine	[203]
Patients with fatigue, anxiety, depression, and low-serum serotonin	SSRIs, SNRIs, NDRIs	[98, 208]
Patients with the immune or allergic phenotype	Antihistamines	[204–207]
Patients with olfactory dysfunction and dysgeusia	Topical nasal steroids	[209]
Those not responding to currently available therapeutics or interested in participating in ongoing research	Many therapeutics are under active investigation	[181]

Abbreviations: COVID-19, coronavirus disease 2019; NDRIs, norepinephrine-dopamine reuptake inhibitors; SNRIs, serotonin-norepinephrine reuptake inhibitors; SSRIs, selective serotonin reuptake inhibitors.

It would be a mistake to finish this section with the impression that we are limited to these few evidence-based interventions. In the evaluation of long COVID, we often diagnose conditions for which we have effective treatments. If a person develops new congestive heart failure, arrhythmia, diabetes, gastroesophageal reflux, POTS, hypertension, renal issues, or a myriad of other medical conditions, we could employ proven therapies for these conditions.

## CONCLUSIONS

The introduction of the novel pathogen SARS-CoV-2 into the human population triggered a response that involved the coordinated efforts of basic scientists, translational scientists, administrators, politicians, and the general public. In September 2021, the NIH was awarded $470 million to perform a national study on the long-term effects of COVID-19.

PASC is a defined syndrome with ongoing, relapsing, or new conditions after documented or diagnosed infection with SARS-CoV-2 with a recognized International Classification of Diseases–10 code, U09.9. There are several recognized biochemical and physiological abnormalities associated with PASC. There are defined risk factors that increase or decrease an individual's risk for developing PASC. There are interventions that can improve this condition. A subset of individuals with PASC meet the criteria for myalgic encephalitis/chronic fatigue syndrome (ME/CFS) and can carry this diagnosis (G93.32) separately or in addition to PASC.

As our understanding of this disease process grows, we will need to remember the foundation for the management of chronic conditions. This includes listening to our patients, validating their experience, sharing the uncertainties around the prognosis, and communicating realistic goals for recovery [19]. Our understanding of factors driving persistent symptoms will continue to grow. The factors associated with the risk for PASC and the rate of resolution of symptoms will continue to be identified. At least for now, the time to complete resolution in an individual patient remains challenging to predict [215].

One big challenge to date and going forward is that there is more disinformation than actual information in this area, as many peddle misinformation for personal gain [216]. For clinicians and patients, COVID-19 is a new disease, as is long COVID. Long COVID is not something clinicians were taught about in medical school. Understanding long COVID has required the efforts of scientists and physician-scientists. The integration of all this knowledge into something that patients can understand and into treatment plans that benefit patients will require the ongoing efforts of scientific clinicians. A scientific clinician is a practicing doctor who stays up to date on the literature, reads the latest published articles with scientific rigor, and advances therapeutics in time with the acquisition of new information. These scientific clinicians must apply the same rigor at the bedside to understand all the new information that their colleagues apply at the bench. This review is just one more step in our attempts to understand this challenging disease.
