# Correction to: Postacute Sequelae of COVID (PASC or Long COVID): An Evidenced-Based Approach

**DOI:** 10.1093/ofid/ofae509

**Published:** 2024-09-05

This is a correction to: Daniel O Griffin, Postacute Sequelae of COVID (PASC or Long COVID): An Evidenced-Based Approach, *Open Forum Infectious Diseases*, Volume 11, Issue 9, September 2024, ofae462, https://doi.org/10.1093/ofid/ofae462.

In the originally published version of this manuscript, the author Ed Yong was erroneously referred to as ‘Ed Young’.

This error has been corrected.

